# ﻿*Fragilariformameireana*, a new araphid diatom species (Bacillariophyta) producing internal valves from Flanders

**DOI:** 10.3897/phytokeys.257.155919

**Published:** 2025-06-09

**Authors:** Bart Van de Vijver, David M. Williams

**Affiliations:** 1 Research Department, Meise Botanic Garden, Nieuwelaan 38, 1860 Meise, Belgium Meise Botanic Garden Meise Belgium; 2 Department of Biology – ECOSPHERE, University of Antwerp, Universiteitsplein 1, 2610 Wilrijk, Belgium University of Antwerp Wilrijk Belgium; 3 Department of Life Sciences, The Natural History Museum, Cromwell Road, London, SW7 5BD, UK The Natural History Museum London United Kingdom

**Keywords:** Belgium, *Fragilariforma*, morphology, new species, resting spores

## Abstract

During a biodiversity survey around the historic Flemish town of Damme, a large *Fragilariforma* taxon was observed in a small pond that could not be identified using the currently available literature. Detailed light and scanning electron microscopy investigations revealed the presence of so-called resting spores within the frustules. Following comparison with similar linear, long-celled *Fragilariforma* species, the unknown taxon is described as new: *Fragilariformameireana***sp. nov.** Discriminating features of the new species include the long, linear valves with protracted, distinctly rostrate apices, the absence of ribbon-like colonies, the presence of a rimoportula on both apices and the formation of internal resting spores.

## ﻿Introduction

The ‘araphid’ genus *Fragilariforma* D.M.Williams & Round was originally described under the name *Neofragilaria* D.M.Williams & Round in the journal *Diatom Research* volume 2(2), which had a printed publication date of December 1987 – the actual publication date was 18^th^ February 1988 (Williams and Round 1987 [1988]). Coincidentally, *Neofragilaria* Desikachary, Prasad & Prema, a marine genus, published in the third volume of their book *Atlas of Diatoms* (Desikachary and Prema 1987), had a printed publication date of 1988 when it was actually published on 15^th^ December 1987 (see Turland et al. 2025, Art. 31.1, ex.3). As *Neofragilaria* D.M.Williams & Round was proved to be a younger homonym of *Neofragilaria* Desikachary, Prasad & Prema, the new name of *Fragilariforma* D.M.Williams and Round (1988) was proposed for the freshwater species, typified by *Fragilariavirescens* (Ralfs) D.M.Williams & Round.

At present, AlgaeBase records 31 accepted species names and 8 accepted varieties in *Fragilariforma* (Guiry and Guiry 2025) and with two further species recently added (Williams 2025) brings the total to 41 taxa (species + varieties). The genus, with *F.virescens* as *typus generis*, is characterised by a very thin to almost absent sternum, uniseriate striae extending onto the mantle composed of small, rounded areolae with simple vela, one rimoportula per valve, well developed, large apical pore fields on each apex, the presence of spines, occasionally linking frustules in long, ribbon-like colonies, and a cingulum composed of 4–6 open, ligulate copulae with a dissected pars interior and a very small septum at the non-ligulate pole.

Species in the genus were already present and diverse in the Miocene (Williams 1996; Wetzel et al. 2013; Williams and Buczkó 2016). Nowadays, *Fragilariforma* has a worldwide distribution with species thriving almost exclusively in freshwater habitats, many of them preferring acidic, oligo-mesotrophic conditions (Round et al. 1990; Almeida et al. 2017).

In Europe, only a handful of species are present with *F.virescens*, *F.bicapitata* (A.Mayer) D.M.Williams & Round, *F.nitzschioides* (Grunow) Lange-Bertalot, and *F.undata* (W.Smith) Heudre, C.E.Wetzel & Ector being the most common (Lange-Bertalot et al. 2017; Van de Vijver et al. 2024). During a biodiversity survey of the fauna and flora around the Flemish city of Damme (Province West-Vlaanderen, Belgium) in 2018, an unusual ‘araphid’ taxon was recorded in a small pool, close to the city. The valves showed all morphological features of the genus *Fragilariforma* and would be best placed within this genus. Peculiarly, almost all observed frustules presented a large internal resting cell, visible as a thick, hyaline rectangular cell, squeezed in between the epivalve and hypovalve.

Resting spores are not uncommon in marine centric diatoms with most *Chaetoceros* species producing resting spores being a good example of such stages (Hargraves and French 1983; McQuoid and Hobson 1996). In pennate diatoms, and more specifically freshwater species, resting stages are less common and it is unclear what purpose they serve. Therefore, we refer to them as internal cells as, morphologically, it describes best their outlook and position. McQuoid and Hobson (1996) presented an overview of all diatom species known to produce either resting spores or resting cells, including a few ‘araphid’ species and members of the genus *Eunotia*. Von Stosch and Fecher (1979) and more recently, Stancheva (2006), analysed the presence of resting stages in *Eunotiasoleirolii* (Kützing) Rabenhorst. The presence of these resting stages also led in the past to the description of *Meridionzinckenii* Kützing (Kützing 1843), now considered a synonym of *M.circulare* (Greville) C.Agardh. Both *Meridion* taxa only differed from each other by the presence of internal cells. Kützing (1843, p. 396) described them as curved lines (Diese Art zeichnet sich dadurch aus, dass zwischen jeder geraden Theilungslinie noch eine gekrümmte vorkommt. Je zwei der gekrümmten Linien vereinigen sich zu einer Ellipse. Die abwechselnden geraden Linien halbiren und trennen die Ellipsen von einander. [This type is characterised by the fact that between each straight dividing line there is a curved one. Any two of these curved lines merge to form an ellipse. The alternating straight lines bisect and separate the ellipses]). The occurrence of internal cells in *Meridion* was also observed by Geitler (1971) and Kociolek et al. (2011) who reported the presence of ‘Innenschalen’ or internal spores in a Chinese *Meridion* population.

The observation of internal cells in the new species of *Fragilariforma* is the first for this genus. Although in *Meridion*, the presence of these cells did not justify the separation of a new species, we believe there are sufficient morphological differences between the new *Fragilariforma* species and all currently known taxa in this genus. The new species is therefore described under the name *Fragilariformameireana* Van de Vijver & D.M.Williams, sp. nov. The species is morphologically characterised using Light (LM) and scanning electron (SEM) microscopy observations. Brief notes on its ecology, based on the associated diatom flora, are added.

## ﻿Material and methods

During the biodiversity survey in the surroundings of the Flemish city of Damme, two samples were collected from a small pool on the corner of Damse Vaart West and Noorweegse Kaai. This pool had a total surface of less than 20 m^2^ and was surrounded by *Phragmites* border. Due to logistic constraints, physicochemical data could not be collected.

One of the collected samples contained a small population of the new *Fragilariforma* species and was prepared for LM and SEM observations following the method described in van der Werff (1955). Small parts of the sub-sample were cleaned by adding 37% H_2_O_2_ and heating to 80 °C for about 1 h, after which the reaction was completed by addition of saturated KMnO_4_. Following digestion and centrifugation (three times 10 minutes at 3700 × rpm), the resulting cleaned diatom material was diluted with distilled water to avoid excessive concentrations of diatom valves on the slide and mounted on permanent slide using Naphrax (refraction index 1.73, Brunel Microscopes, UK ltd.). The resulting slides were analysed using an Olympus BX53 microscope at 1000× magnification (UPlan FL N 100× oil objective, N.A. 1.30), equipped with Differential Interference Contrast (Nomarski) optics and the Olympus UC30 Imaging System connected to the Cell Sense Standard program. The number of specimens, measured at random on the type slide, is indicated (n = X). An ecological characterisation of the new species is added based on the accompanying diatom flora, assessed by counting 400 diatom valves along random transects. Relative abundances, when given, are expressed as a percentage of counted valves.

For SEM, part of the suspension was filtered through 5-μm Isopore™ polycarbonate membrane filters (Merck Millipore), pieces of which were fixed on aluminium stubs after air–drying and coated with a platinum layer of 20 nm, and studied using a JEOL-JSM-7100F field emission scanning electron microscope at 2 kV. Slides, samples and stubs are stored at the BR-collection (Meise Botanic Garden, Belgium). Plates were prepared using Photoshop CS5.

Terminology used in the description of the various structures of the siliceous cell wall is based on Williams and Round (1987 [‘1988’], for *Fragilariforma* [=former Neofragilaria D.M.Williams & Round] genus features) and Round et al. (1990, for *Fragilariforma* genus features). For taxonomic comparisons, the following papers were consulted: Hustedt (1932), Mayer (1917, 1937), Krammer and Lange-Bertalot (1991), Kingston et al. (2001), Kilroy et al. (2003), Metzeltin and Lange-Bertalot (2007), Wetzel et al. (2013), Lange-Bertalot et al. (2017), Almeida et al. (2017), Pottiez et al. (2024), Van de Vijver et al. (2024), and Williams (2025).

**Figure 1.**
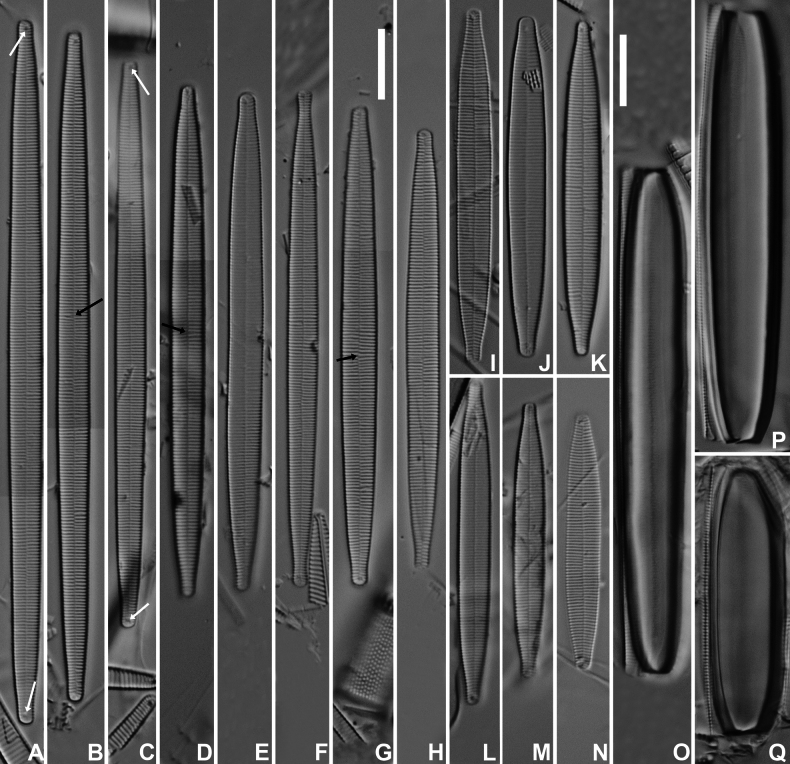
*Fragilariformameireana* Van de Vijver & D.M.Williams, sp. nov., LM micrographs taken from the holotype material (BR-4867, Damme, Belgium) **A–N**LM pictures of valves in valve face view in decreasing length. The white arrows indicate the rimoportulae. The black arrows indicate anomalies in the striation pattern **O–Q**LM pictures of three frustules showing internal cells. Scale bars: 10 µm.

**Figure 2.**
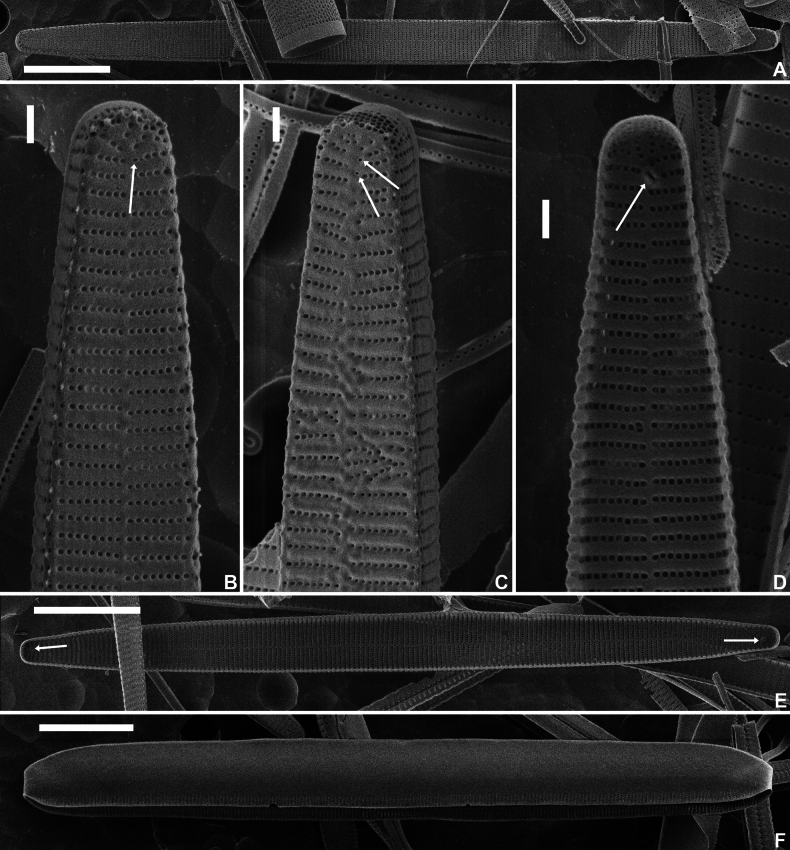
*Fragilariformameireana* Van de Vijver & D.M.Williams, sp. nov., SEM micrographs taken from the holotype material (BR-4867, Damme, Belgium) **A**SEM external view of an entire valve **B, C**SEM external detail of two valve apices showing the rimoportulae (white arrows) and the apical pore field. Note the rudimentary spines and the irregular striation pattern on **C. D**SEM internal detail of a valve apex showing the rimoportula (white arrow) **E**SEM internal view of an entire valve. The rimoportulae are indicated with white arrows. **F**SEM view of the broad valvocopula. Scale bars: 10 µm (**A, E–F**); 1 µm (**B–D**).

**Figure 3.**
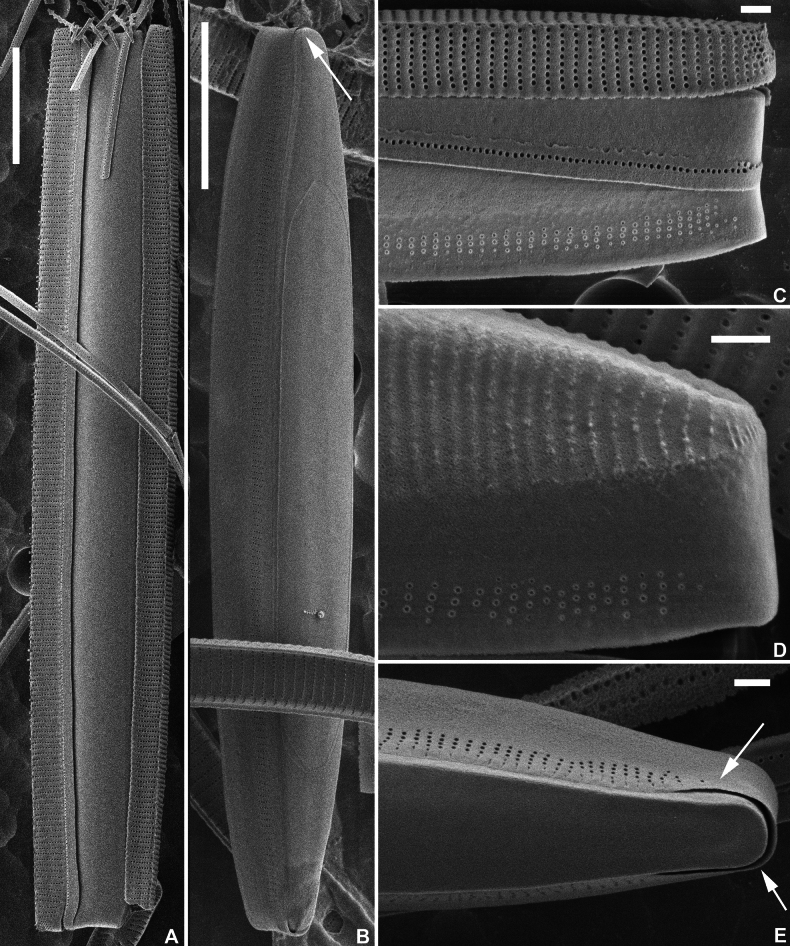
*Fragilariformameireana* Van de Vijver & D.M.Williams, sp. nov., SEM micrographs taken from the holotype material (BR-4867, Damme, Belgium) **A**SEM view of a complete frustule with the internal cell inserted between the valves **B**SEM view of the resting spore covered by the valvocopula. At the apices, the valvocopula is well visible. Occasionally, very shallow ridge present on the plain side visible as a thin line. **C**SEM external detail of the valve apex with the valve, internal cell and one detached copula **D**SEM external side view of the internal cell. Note the ridges on the top **E**SEM external valve face view of the internal cell surrounded by the valvocopula. Scale bars: 10 µm (**A, B**); 1 µm (**C–E**).

For typification of the species, we chose to use the entire slide as the type, following article 8.2 of the International Code of Nomenclature for algae, fungi, and plants (Turland et al. 2025). Diatoms show a broad variability along their cell cycle making the choice for the entire population on the slide more obvious, but because of admixtures, one valve was indicated to best illustrate the taxon (see Figs 1–3). All novelties are registered proactively according to Art. 42.3 (Turland et al. 2025).

## ﻿Results

### ﻿ Fragilariforma meireana

Taxon classificationPlantaeFragilarialesFragilariaceae

Van de Vijver & D.M.Williams sp. nov.

3C2E4075-96BC-5BE5-8863-7A6DD77185F6

Figs 1 , 2 , 3

#### Holotype.

BR-4867 (Meise Botanic Garden, Belgium). Fig. 1B represents the holotype.

#### Isotype.

Slide 453 (University of Antwerp, Belgium).

#### Registration.

http://phycobank.org/105490.

#### Type locality.

Unnamed pool at corner of Damse Vaart West and Noorweegse Kaai (Damme, West-Vlaanderen, Belgium), sample DAMME-6, (coll. date 26.v.2018, leg. B. Van de Vijver).

#### Etymology.

This species is named in honour of Prof. Dr Patrick Meire, former head of the research group ECOBE (Department of Biology, Antwerp University, Belgium), and initiator of the biodiversity survey in Damme in 2018.

#### Description.

***LM*** (Fig. 1). Valves linear with almost parallel, straight valve margins only very slightly narrowing just before the valve apices. Apices clearly protracted, distinctly rostrate, very rarely capitate (Fig. 1F). Valve dimensions (n=25): length 35–100 µm, width 4–5 µm. Sternum very narrow, linear, running from apex to apex. Central area absent. Striae parallel throughout the entire valve length, becoming very weakly radiate at the apices, 22–25 in 10 µm. Occasionally, two stria diverging near the valve centre (Fig. 1B, D, G, black arrows). Rimoportula visible at both apices (Fig. 1A, C, white arrows). Internal cells large, hyaline, robust (Fig. 1O–Q). ***SEM*** (Figs 2, 3). Valve face flat with slightly raised virgae (Fig. 2A, C). Striae uniseriate, composed of a series of small, rounded areolae (Fig. 2B, C). Small marginal spines present, irregularly placed on the valve face/mantle junction (Fig. 2B), usually on the virgae. Sternum very narrow, slightly raised (Fig. 2B). Apical porefield large, entirely on the mantle, composed of at least 3–4 rows of round to quadratic porelli (Fig. 2C). Rimoportula very small, externally rimmed (Fig. 2B, white arrows). Occasionally, 2 rimoportulae present at one pole (Fig. 2C, white arrows). Internally rimoportula small, weakly oblique (Fig. 2D, E, white arrows). Valvocopula very large, plain on one side and with series of pores on the other (Fig. 2F). Copulae small, with serrated edge, bearing one row of pores (Fig. 3C). Internal cell robust (Fig. 3A, B), located between both valves. Valvocopula covering most of the internal cell (Fig. 3B, E, arrows). Internal cell with irregular ridged ornamentation (Fig. 3D) on one side, plain on the other (Fig. 3E). Occasionally, very shallow ridge present on the plain side visible as a thin line (Fig. 3B).

#### Associated diatom flora and ecology.

*Fragilariformameireana* has so far only been found in the small pool in Damme. The diatom flora in the pool is dominated by *Eunotiabilunaris* (Ehrenberg) Mills *s.l.* (21% of all counted valves), *Aulacoseiraitalica* (Ehrenberg) Simonsen (19.5%), *Fragilariacampyla* (Hilse) Van de Vijver *et al.* (17%), *Nitzschiaarchibaldii* Lange-Bertalot (8%) and *Gomphonemautae* Lange-Bertalot & Reichardt (6%). Most of these species are known to prefer meso- to eutrophic conditions with moderate to higher electrolyte contents, circumneutral to alkaline conditions (Lange-Bertalot et al. 2017). The presence of *Eunotiabilunaris* in this community is somewhat unusual as the species is mostly known to prefer oligotrophic, oligosaprobic conditions. However, it is clear that *E.bilunaris* as we know it today represents a complex of taxa present in different environmental conditions (Vanormelingen et al. 2008).

## ﻿Discussion

The position of *Fragilariformameireana* within the genus *Fragilariforma* is justified by the presence of apical porefields, rimoportulae on both apices, a very narrow sternum, uniseriate striae and the presence of marginal spines, although they are not used to link frustules to form ribbon-like colonies (Williams and Round 1987 [1988]).

The number of morphological features to separate *Fragilariforma* species is limited and mostly based on valve outline, striation pattern and spine structure. Most *Fragilariforma* species currently known have either constricted or inflated valves and do not show long, linear valves in their cell cycles, such as *F.undata* (W.Smith) Heudre et al., *F.cassieae* Kilroy & E.Bergey, or *F.floridana* (Hanna) D.M.Williams (Williams 1990; Kilroy et al. 2003; Metzeltin and Lange-Bertalot 2007; Van de Vijver et al. 2024). Several *Fragilariforma* species have similar long, linear valves with parallel margins, although none of them show the combination of features observed in *F.meireana*, such as the presence of internal cells, two rimoportulae and rudimentary spines. In the European flora, longer *Fragilariforma* valves were included within *F.virescens*, occasionally given different names such as Fragilariavirescensvar.birostrata A.Mayer, described in 1917. Reid et al. (1995) discussed the presumable cosmopolitan nature of *F.virescens* stating that this may be misleading given that many morphologically different taxa were included within *F.virescens*, incorrectly broadening its original description. Pottiez et al. (2024) investigated the type material of *F.virescens* but did not find valves longer than 38 µm. This maximum length of 38 µm may be an underestimation of the length the species can reach as it represents only one population and it is generally accepted that longer valves can be found (Williams and Round 1987 [1988]; Williams 2001). Analysis of published illustrations of *F.virescens* in Europe, however, showed that none of the illustrated *F.virescens* valves seem to be represented by *F.meireana*. Mayer (1917, 1937) illustrated a large number of F.virescens valves including his var. birostrata but all valves have a different valve outline compared to *F.meireana* with truncated, only shortly protracted, broadly rounded apices. Cleve-Euler (1953, fig. 361) shows longer *F.virescens* valves but always with very protracted, capitate apices, a feature not observed in *F.meireana* that has rostrate, rarely capitate apices. Another *Fragilariforma* species with linear valves is *F.nitzschioides* (Grunow) Lange-Bertalot but this species forms long, ribbon-like colonies with strongly linked frustules bearing thick, spatulate spines. Moreover, *F.nitzschioides* has broadly rounded but not protracted apices whereas *F.meireana* has cuneately formed apices (Krammer and Lange-Bertalot 1991, plate 128, figs 1–10).

In the southern hemisphere, several longer *Fragilariforma* species have been described, but almost all of these species form long, ribbon-like colonies using well-developed marginal linking spines, a feature not observed in *F.meireana*. Wetzel et al. (2013) discussed the morphology of *F.javanica* (Hustedt) C.E.Wetzel, E.Morales & Ector, a colony-forming, linear-shaped species with clearly capitate apices. Similarly, *Fragilariformasiamensis* (Østrup) D.M.Williams (Williams 2025) and *F.telum* (J.R.Carter & Denny) P.D.Almeida, C.E.Wetzel & E.Morales (Almeida et al. 2017) show elongated, linear valves with capitate apices and a weak constriction near the valve centre, separating these species from *F.meireana*.

It is possible that populations of *F.meireana* have been mistakenly identified in the past as *Ulnaria* or *Fragilaria* taxa but the very fine striation, the very thin sternum and the absence of a central area are features that are very rarely seen in these two genera, making it difficult to find comparable taxa in these two genera.

The presence of the internal cells is somewhat surprising in this genus. In the sample, it is the only species to present such cells. None of the other recorded taxa show any sign of internal cells, teratologies or any other form of anomaly. As stated in the introduction, there are several ‘araphid’ and brachyraphid taxa showing such internal cells including *Eunotiasoleirolii* and *Meridioncirculare* (as *M.zinckenii*). Von Stoch and Fecher (1979) discussed the formation of what they called ‘resting spores’ in *E.soleirolii* indicating important differences with similar internal cells in centric diatoms. In pennate diatoms, these internal cells are formed in frustules keeping their girdle bands, as seems to be also the case in *F.meireana*. In their research, von Stosch and Fecher (1979, p. 236) indicated several environmental condition that seem to induce the formation of these cells, including nutrient and silica deficiencies, but it is unclear what has caused the formation of these internal cells in *F.meireana*.

At present, only one population of *F.meireana* has been found, but given the very distinct morphological features of the observed valves, the separation of *F.meireana* as a new species is justified.

## Supplementary Material

XML Treatment for Fragilariforma meireana

## ﻿Additional information

### Author ORCIDs

Bart Van de Vijver https://orcid.org/0000-0002-6244-1886

David M. Williams https://orcid.org/0000-0002-0584-307X

### Data availability

All of the data that support the findings of this study are available in the main text.

## References

[B1] Almeida PD Wetzel CE Morales EA Ector L Bicudo DC (2017) New species and combinations on *Fragilariforma* (Bacillariophyta) from tropical freshwater environments. Fottea 17(2): 277–292. 10.5507/fot.2017.006

[B2] Cleve-Euler A (1953) Die Diatomeen von Schweden und Finnland. Teil II. Arraphideae, Brachyraphideae. Kungliga Svenska Vetenskapsakademiens Handlingar, ser. IV 4(1): 1–158.

[B3] Desikachary TV Prema P (1987) Diatoms from the Bay of Bengal. Atlas of Diatoms. Fasc. III. Madras Science Foundation, Madras, 1–10. [pls. 222–331]

[B4] Geitler L (1971) Die inäquale Teilung bei der Bildung der Innenschalen bei *Meridioncirculare*. Österreichische. Botanicheskii Zhurnal 119(4/5): 442–446. 10.1007/BF01377496

[B5] Guiry MD Guiry GM (2025) AlgaeBase. World-wide electronic publication, University of Galway. https://www.algaebase.org [searched on April 8, 2025]

[B6] Hargraves P French F (1983) Diatom resting spores: significance and strategies. In: Fryxell G (Ed.) Survival Strategies of the Algae.Cambridge University Press, New York, 49–58.

[B7] Hustedt F (1932) Die Kieselalgen Deutschlands, Österreichs und der Schweiz unter Berücksichtigung der übrigen Länder Europas sowie der angrenzenden Meeresgebiete. Vol. VII. Teil 2. Liefrung 2. Rabenhorst’s Kryptogamen Flora von Deutschland, Österreich und der Schweiz. Leipzig, 177–320.

[B8] Kilroy C Sabbe K Bergey EA Vyverman W Lowe R (2003) New species of *Fragilariforma* (Bacillariophyceae) from New Zealand and Australia. New Zealand Journal of Botany 41: 535–554. 10.1080/0028825X.2003.9512868

[B9] Kingston JC Sherwood AR Bengtsson R (2001) Morphology and taxonomy of several *Fragilariforma* taxa from Fennoscandia and North America. 16^th^ International Diatom Symposium, 25 Aug. – 1 Sept. 2000, 73–88.

[B10] Kociolek JP Liu Y Wang Q (2011) Internal valves in populations of *Meridioncirculare* from the A’er Mountain region of northeastern China: Implications for taxonomy and systematics. Journal of Systematics and Evolution 49(5): 486–494. 10.1111/j.1759-6831.2011.00142.x

[B11] Krammer K Lange-Bertalot H (1991) Bacillariophyceae, 3. Teil: Centrales, Fragilariaceae, Eunotiaceae. Spektrum Akademischer Verlag, Heidelberg, 1–578.

[B12] Kützing FT (1843) Ueber Meridion Zinckeni. Botanische Zeitung 1(23): 396.

[B13] Lange-Bertalot H Hofmann G Werum M Cantonati M (2017) Freshwater benthic diatoms of Central Europe: over 800 common species used in ecological assessment. English edition with updated taxonomy and added species. Koeltz Botanical Books, Schmitten-Oberreifenberg, 1–942.

[B14] Mayer A (1917) Beiträge zur Diatomeenflora Bayerns. Part I, A. Bacillariales aus dem Fichtelgebirge und angrenzenden Gebieten. B. Bacillariales aus dem Bayrischen Walde. Denkschriften der Königlich-Bayerischen Botanischen Gesellschaft in Regensburg 13: 1–99.

[B15] Mayer A (1937) Die Bacillariophyten-Gattungen Fragilaria und Asterionella in Bayern Berichte der Bayerischen Botanische Gesellschaft 22: 50–85.

[B16] McQuoid M Hobson L (1996) Review: Diatom resting stages. Journal of Phycology 32: 889–902. 10.1111/j.0022-3646.1996.00889.x

[B17] Metzeltin D Lange-Bertalot H (2007) Tropical diatoms of South America II. Special remarks on biogeography disjunction. Iconographia Diatomologica 18: 1–877.

[B18] Pottiez M Williams DM Van de Vijver B (2024) Observations on the lectotype material of *Fragilariformavirescens* (Fragilariaceae, Bacillariophyceae). Notulae Algarum 321: 1–7.

[B19] Reid G Huxley R Williams DM (1995) Preliminary efforts towards a diatom type catalogue with an example from *Fragilariformavirescens*. In: Marino D Montresor M (Eds) Proceedings of the 13th International Diatom Symposium.BioPress Ltd, 423–429.

[B20] Round FE Crawford RM Mann DG (1990) The diatoms biology and morphology of the genera. Cambridge University Press, 1–747.

[B21] Stancheva R (2006) Distribution of resting spores of *Eunotiasoleirolii* and Meridioncircularevar.constrictum (Bacillariophyta) in sediments of peat bogs from Mt. Central Sredna Gora, Bulgaria In: Ognjanova-Rumenova N, Manoylov K (Eds) Advances in Phycological Studies, Pensoft, Sofia and Moscow, 111–121.

[B22] Turland NJ Wiersema JH Barrie FR Gandhi KN Gravendyck J Greuter W Hawksworth DL Herendeen PS Klopper RR Knapp S Kusber W-H Li D-Z May TW Monro AM Prado J Price MJ Smith GF Señoret CZ (2025) International Code of Nomenclature for algae, fungi, and plants (Madrid Code). The University of Chicago Press, Chicago.

[B23] Van de Vijver B Heudre D Wetzel CE (2024) Lectotypification of *Fragilariformaundata* (W.Smith) Heudre & al. (Fragilariaceae, Bacillariophyceae). Notulae Algarum 336: 1–9.

[B24] van der Werff A (1955) A new method of concentrating and cleaning diatoms and other organisms. Verhandlungen der internationalen Vereinigung für theoretische und Angewandte Limnologie 12(1): 276–277. 10.1080/03680770.1950.11895297

[B25] Vanormelingen P Chepurnov V Mann DG Vyverman W (2008) Genetic divergence and reproductive barriers among morphologically heterogeneous sympatric clones of *Eunotiabilunaris* sensu lato (Bacillariophyta). Protist 159(1): 73–90. 10.1016/j.protis.2007.08.004 17964215

[B26] von Stosch HA Fecher K (1979) “Internal thecae” of *Eunotiasoleirolii* (Bacillariophyceae): Development, structure and function as resting spores. Journal of Phycology 15(3): 233–243. 10.1111/j.0022-3646.1979.00233.x

[B27] Wetzel CE Morales EA Hinz F Bicudo DC Ector L (2013) *Fragilariformajavanica* comb. nov.: Analysis of type material of a widely reported species with a tropical distribution. Diatom Research 28(4): 373–379. 10.1080/0269249X.2013.809668

[B28] Williams DM (1990) *Fragilariafloridana* Hanna: Ultrastructure of the valve and girdle and its transference to *Fragilariforma* Williams & Round. Ouvrage dédié à la Mémoire du Professeur Henry Germain (1903–1989). Koeltz Scientific Books, Königstein, 259–265.

[B29] Williams DM (1996) Notes on the genus *Fragilariforma* (Fragilariophyceae: Bacillariophyta) with a description of a new Miocene fossil species, *Fragilariformaplatensis*. Beihefte zur Nova Hedwigia 112: 283–288.

[B30] Williams DM (2001) Comments on the structure of “post-auxospore” valves of *Fragilariformavirescens* In: Jahn R, Kociolek JP, Witkowski A, Compère P (Eds) Lange-Bertalot-Festschrift: Studies on Diatoms. Dedicated to Prof. Dr Dr h.c. Horst Lange-Bertalot on the occasion of his 65^th^ Birthday. A.R.G. Gantner Verlag, 103–117.

[B31] Williams DM (2025) On the type specimens of *Fragilariasiamensis* Østrup, its transfer to *Fragilariforma* D.M. Williams & Round, and comments on the biogeography of the genus. Diatom Research 40(1): 21–27. 10.1080/0269249X.2024.2412852

[B32] Williams DM Buczkó K (2016) *Fragilariforma Hajósiae*: Re-description and revision of Pantocsek’s species *Diatoma fossile* (Bacillariophyta). Phytotaxa 244(2): 181–190. 10.11646/phytotaxa.244.2.6

[B33] Williams DM Round FE (1987 [1988]) Revision of the genus *Fragilaria*. Diatom Research 2(2): 267–288. 10.1080/0269249X.1987.9705004

[B34] Williams DM Round FE (1988) *Fragilariforma*, nom. nov., a new generic name for *Neofragilaria* Williams & Round. Diatom Research 3: 265–266. 10.1080/0269249X.1988.9705039

